# Identification of Genes Associated with Liver Metastasis in Pancreatic Cancer Reveals PCSK6 as a Crucial Mediator

**DOI:** 10.3390/cancers15010241

**Published:** 2022-12-30

**Authors:** Hang He, Shuman Zhang, Hai Yang, Pengyan Xu, Isabella Kutschick, Susanne Pfeffer, Nathalie Britzen-Laurent, Robert Grützmann, Deliang Fu, Christian Pilarsky

**Affiliations:** 1 Department of Surgery, Universitätsklinikum Erlangen, Friedrich-Alexander Universität Erlangen-Nürnberg (FAU), 91054 Erlangen, Germany; 2 Department of Pancreatic Surgery, Huashan Hospital, Shanghai Medical College, Fudan University, Shanghai 200040, China

**Keywords:** CRISPR/Cas9, PCSK6, liver metastasis, ERK

## Abstract

**Simple Summary:**

Liver metastasis is the most frequent event of tumor progression in pancreatic cancer, and available therapeutics have achieved unsatisfactory outcomes. The underlying molecular mechanisms of liver metastasis in pancreatic cancer remain unclear and critical genes participating in this process will provide new therapeutic targets. We identified the liver-metastasis-related genes via bioinformatics analysis of the molecular profiling of liver metastatic lesions in pancreatic cancer. Using CRISPR/Cas9 technology, we investigated the biological functions and molecular mechanism of PCSK6 involved in pancreatic cancer. Our findings revealed that PCSK6 inactivation could suppress liver metastasis efficiently, and PCSK6 might serve as a novel molecular target for improving the therapeutic efficacy of liver metastasis in pancreatic cancer.

**Abstract:**

Liver metastasis occurs frequently in patients with pancreatic cancer. We analyzed the molecular profiling in liver metastatic lesions aiming to uncover novel genes responsible for tumor progression. Bioinformatics analysis was applied to identify genes directing liver metastasis. CRISPR/Cas9 technology was used to knock out the candidate gene. Proliferation assays, colony formation assays, cell cycle analysis, migration assays, wound healing assays, Immunofluorescence analysis, and the tumor xenograft model of intrasplenic injection were adopted to evaluate the effects of PCSK6 inactivation on cell growth, migration and liver metastasis. GSEA and Western blot were used to investigate the corresponding signaling pathway. PCSK6 was one of the obtained liver-metastasis-related genes in pancreatic cancer. PCSK6 inactivation inhibited cell growth and cell migration, due to G0/G1 cell cycle arrest and the remodeling of cell–cell junctions or the cell skeleton, respectively. PCSK6 inactivation led to fewer counts and lower outgrowth rates of liver metastatic niches in vivo. The Raf-MEK1/2-ERK1/2 axis was repressed by PCSK6 inactivation. Accordingly, we found PCSK6 inactivation could inhibit cell growth, cell migration, and liver metastasis, and explored the role of the Raf-MEK1/2-ERK1/2 axis in PCSK6 inactivation. PCSK6-targeted therapy might represent a novel approach for combatting liver metastasis in pancreatic cancer.

## 1. Introduction

Pancreatic cancer has been identified as one of the most aggressive malignant tumors in the human alimentary system and the 5-year survival rate of patients with pancreatic cancer has been found to be approximately 11% [1]. Fewer than 20% of the patients with pancreatic cancer are candidates for curative resections of tumors, whereas most of the patients are found to have unresectable disease or distant metastasis, of which liver metastasis account for 50% [2]. Even among patients who undergo curative operations, 80% of cases experience recurrence within 2 years and the liver is the most frequently involved organ postoperatively [3,4]. Unfortunately, applicable therapies such as systemic chemotherapy, stereotactic body radiation therapy, transarterial chemoembolization, radiofrequency ablation, cryoablation and ethanol injection, have achieved unsatisfactory effects on liver metastasis in current clinical practice [5]. There is an imperative need to respond this challenge by developing innovative targets in treatments of liver metastasis in pancreatic cancer.

In previous studies, multiple novel molecular targets with respect to liver metastasis had been found in human malignancies [6,7,8,9,10], which improved patients’ outcomes and highlighted the roles of molecular targets in liver metastasis [1]. Clinical trials combining different molecular inhibitors with chemotherapies in patients with local advanced or metastatic disease of pancreatic cancer are ongoing, although initial results are discouraging [11]. Due to transitions in the tumor microenvironment (TME) from primary tumors to liver metastasis and corresponding variations in the molecular profiling, distinctive molecular targets found in liver metastasis may provide active areas of research and clinical benefits. Several studies have made some breakthrough in these fields [12,13], but no doubt more attempts will be expected.

The crosstalk between tumors cells and the TME, has been posited to induce dynamic variations in molecular profiling, and therefore we attempted to identify differentially expressing genes (DEGs) in liver metastasis compared to primary tumors of pancreatic cancer. Out of 29 candidate genes, proprotein convertase subtilisin/kexin type 6 (PCSK6), was found to be an up-regulated DEG in liver metastasis in this study, and was closely related to tumor progression [14]. Current evidence has shown that PCSK6 participates in proliferation, migration and apoptosis of tumor cells in different human malignancies [15,16], but its role in the liver metastasis of pancreatic cancer remains unclear.

In this study, we used CRISPR/Cas9 technology to knock out PCSK6 in pancreatic cancer in vitro and in vivo, aiming to explore the functions of PCSK6 and the corresponding mechanism involved in liver metastasis, to update the knowledge regarding the role of PCSK6-targeted therapeutics in the treatments of the liver metastasis in pancreatic cancer.

## 2. Materials and Methods

### 2.1. Bioinformatics Analysis of Liver-Metastasis-Related Genes

Transcriptome data were collected from Gene Expression Omnibus (GEO, www.ncbi.nlm.nih.gov/geo/, accessed on 15 August 2021). The searching criteria were defined as pancreatic cancer OR pancreatic tumor OR pancreatic ductal adenocarcinoma (keyword), Homo sapiens (organisms), and expression profiling by array (study type).

DEGs were generated between liver metastasis and primary tumor in each data series, respectively using the online tool GEO2R (www.ncbi.nlm.nih.gov/geo/geo2r, accessed on 15 August 2021), with parameters including adjusted *p*-values and the Benjamini Hochberg (false discovery rate). Log transformation was applied to the data. DEGs with Log2 FC ≥ 1 or ≤−1 and adjusted *p*-values < 0.05 were considered statistically significant. Overlapping DEGs from different data series were obtained using Venn diagrams (bioinformatics.psb.ugent.be/webtools/Venn, accessed on 29 August 2021).

Overlapping DEGs were validated in The Cancer Genome Atlas (TCGA) and Genotype-Tissue Expression (GTEx) database using an online tool GEPIA2 (gepia2.cancer-pku.cn/#index, accessed on 10 September 2021). Adjusted *p*-values < 0.01 and Log2 FC ≥ 1 or ≤−1 were defined as cutoff values.

To exclude the influence of mesenchymal components, DEGs obtained in the previous step were verified in cell lines of pancreatic cancer using Cancer Cell line Encyclopedia (CCLE) through an online tool DepMap Portal (depmap.org/portal/, accessed on 12 September 2021). The mRNA expression profiles of 13 cell lines from CCLE (ASPC1, BXPC3, CAPAN1, CAPAN2, CFPAC1, HPAC, HPAFII, HS766T, MIAPACA2, PANC1, SU8686, SUIT2 and SW1990) were applied. The mRNA expression of each gene was presented as transcripts per kilobase million (TPM). Log2(TPM + 1) < 2 was defined as negative expression. The DEGs in the previous step with Log2(TPM + 1) < 2 in all 13 cell lines were excluded. The verified DEGs were represented by heat plots using an online tool Heatmapper (www.heatmapper.ca/, accessed on 13 September 2021). Lasso Cox regression (R package glmnet, R version 4.2.1) was performed, followed by univariate and multivariate Cox regression (R package survival, R version 4.2.1), to find prognostic genes among the candidate genes generated above. For overall survival analysis, cohorts were grouped into high and low expressions of DEGs using the median expressions of candidate genes in the TCGA database. Kaplan–Meier survival plots were drawn, and Log-rank tests were employed (R package survminer, R version 4.2.1). The cutoff of *p*-values was defined as <0.05.

### 2.2. Cell Culture

The human pancreatic cancer cell line SUIT-2 was obtained from the Japanese Collection of Research Bioresources Cell Bank (JCRB1094, RRID: CVCL_3172) and cultured in minimum essential medium (Gibco, 31095-029) supplemented with 10% fetal bovine serum (Gibco, A3160801). Another human pancreatic cell line MIA PaCa-2 was obtained from ATCC (CRM-CRL-1420, RRID: CVCL_0428) and cultured in Dulbecco’s modified Eagle’s medium (Gibco, 31966-021) supplemented with 10% fetal bovine serum and 2.5% horse serum (Gibco, 16050-130). Both cell lines were incubated in a humidified atmosphere with 5% CO_2_ at 37 °C.

### 2.3. CRISPR/Cas9 Gene Editing

In this study, PCSK6 was knocked out in the cell lines mentioned above using the CRISPR/Cas9 gene editing system as described previously [17]. Briefly, forward and reverse strands of oligonucleotides for the designed sgRNAs targeting PCSK6 were synthesized by Eurofins Genomics (Table S1). The sgRNA oligos were cloned into the pSpCas9(BB)-2APuro (PX459) V2.0 vector, which was kindly provided by Feng Zhang (Addgene #62988, http://n2t.net/addgene:62988, RRID: Addgene_62988, accessed on 14 September 2021). The vectors with inserted sgRNA oligos were transformed into Endura DUOs Electrocompetent Cells (BioSEARCH Technologies, 60242-2, Middleton, WI, USA). The CRISPR plasmids were isolated and validated via DNA sequencing. Cell lines were transfected with the validated CRISPR plasmids using a Lipofectamine 3000 Transfection Kit (Invitrogen, L3000-008, Waltham, MA, USA) according to the manufacturer’s instructions. Puromycin (Invivogen, ant-pr-1, 10 μg/mL, Waltham, MA, USA) was used to eliminate un-transfected cells 48 h after transfection and the generated pool cells were seeded in a 96-well plate to establish single clones. The finally generated cells included SUIT-WT (SUIT-2 wild type), SUIT-NC (negative control transfected with nonsense sgRNA), SUIT-SC1 (single clone with knockout of PCSK6 by sgRNA1), SUIT-SC2 (single clone with knockout of PCSK6 by sgRNA2), MIA-WT (MIA PaCa-2 wild type), MIA-NC (negative control transfected with nonsense sgRNA), MIA-SC1 (single clone with knockout of PCSK6 by sgRNA1) and MIA-SC2 (single clone with knockout of PCSK6 by sgRNA2). RT-PCR and genomic DNA sequencing were both performed to validate the single clones.

### 2.4. RT-PCR

Quantitative analysis of PCSK6 mRNA was performed by means of RT-PCR. Briefly, total RNAs were extracted from cells using a NucleoSpin RNA Plus Kit (MACHEREY-NAGEL, 740984.250, Dueren, Germany). The concentration and purity of the obtained RNAs were determined using the NanoDrop 2000 Spectrophotometer. RNAs were converted to cDNAs using a High Capacity cDNA Reverse Transcription Kit (Applied Biosystems, 4368814, Baltics, Vilnius, Lithuania). Real time PCR was performed via the Bio-Rad CFX Connect Real-Time System, using a PowerSYBR Green PCR Master Mix kit (Applied Biosystems, 4367659, Woolston Warrington, UK) following the manufacturer’s instructions. 2^−∆∆ct^ values were calculated for the relative quantification of mRNAs. Forward and reverse primers for real-time PCR were synthesized by Eurofins Genomics (Ebersberg, Germany, Table S1).

### 2.5. Construction of Plasmid for Genomic DNA Sequencing

Genomic DNA was isolated using a NucleoSpin Kit (MACHEREY-NAGEL, 740952.250, Dueren, Germany). Amplifications of sgRNAs targeting regions in the obtained genomic DNA were performed with Q5^®^ Hot Start High-Fidelity 2X Master Mix (New England Biolabs, M0494L, Frankfurt, Germany) according to the manufacturer’s instructions and primers for sgRNAs targeting regions were synthesized by Eurofins Genomics (Table S1). Products of DNA amplifications were purified with Wizard^®^ SV Gel and a PCR Clean-Up System (Promega, A9281, Madison, WI, USA), followed by ligation of the inserted DNA and vector using NEB PCR Cloning Kits (New England Biolabs, E1203S, Frankfurt, Germany). The generated vectors in above step were transformed into Competent E. coli (New England Biolabs, C3019, Frankfurt, Germany) and isolated using GeneJET Plasmid miniprep Kit (Thermo Scientific, K0503, Baltics UAB, Vilnius, Lithuania). DNA Sequencing of the obtained plasmids was performed by Eurofins Genomics.

### 2.6. Proliferation Assay

Cells were seeded on a 96-well plate (1000 cells/well) and stained with DAPI (Invitrogen, H3570, Eugene, OR, USA) in quintuplicate at 6 h, 24 h, 48 h, 72 h, 96 h and 120 h after seeding, respectively. Images were obtained using an Evos FL Auto 2 imaging system (Invitrogen by Thermo Fisher Scientific, Bothell, WA, USA). Counts of cells were determined using Thermo Scientific HCS Studio Cellomics Scan software (Version 6.60-1.00x, build 8153) and ratios of cell proliferation were calculated using the following formula: mean of cell counts (N hours)/mean of cell counts (6 h).

### 2.7. Colony Formation Assay

Cells were seeded on 6-well plates (100 cells/well) in triplicate and incubated for 8 days. Then, the medium was removed, and cells were fixed with 4% formaldehyde solution for 20 min. Cells were stained with 0.1% crystal violet for 15 min. Colonies consisting of more than 50 cells were counted visually and mean counts of colonies were calculated.

### 2.8. Cell Cycle Analysis

A total of 10^6^ cells in the exponential phase were collected and fixed in 70% cooled ethanol for 30 min. Cells were incubated with Propidium Iodide Solution (Biolegend, 421301, München, Germany) and RNse A (MACHEREY-NAGEL, R2045S, Dueren, Germany) for 15 min at room temperature, followed by adding cooled PBS to terminate the reaction. Stained cell samples were examined by means of flow cytometry (BD Biosciences LSRII, San Jose, CA, USA) and data were processed using FlowJo v10.8 Software (BD Life Sciences, Ashland, OR, USA).

### 2.9. Migration Assay

After starving for 24 h, 5 × 10^4^ cells were seeded in superior chamber of 8.0 μm Corning FluoroBlok Cell Culture Insert (Corning, 351152, Durham, NC, USA) adding medium without fetal bovine serum, whereas medium with 10% fetal bovine serum was added to the inferior chamber. Cells on the inferior surface of the superior chamber were stained with DAPI (Invitrogen, H3570) 20 h after seeding and images were obtained using an Evos FL Auto 2 imaging system (Invitrogen by Thermo Fisher Scientific, Bothell, WA, USA). Counts of migrated cells in each field (30 fields in total) were determined using Thermo Scientific HCS Studio Cellomics Scan software (Version 6.60-1.00x, Build 8153) and mean migrated cells were calculated according to the following formula: cell counts per field = total counts/30 fields.

### 2.10. Wound-Healing Assay

In the wound-healing assay, 10^6^ cells were seeded in 6-well plates and starved in medium without fetal bovine serum before drawing a straight line in each well to establish the wound. Images were obtained at 6 different regions of the wound in each well, at baseline and 20 h after establishing the wound, using an Evos FL Auto 2 imaging system (Invitrogen by Thermo Fisher Scientific, Bothell, WA, USA). The areas of the wounds in the 6 above mentioned regions of each well were determined using imageJ (1.53 k, National Institute of Health, Bethesda, MD, USA). The ratios of wound change (healing ratio) were calculated using the following formula: (area of region ^baseline^–area of region ^20 h^)/area of regions ^baseline^.

### 2.11. Immunofluorescence

In this step, 10^5^ Cells were seeded on cell culture slides (SPL Life Sciences, 30104, Gyeonggi-do, Korea) and fixed with 4% formaldehyde solution, followed by permeabilization with 0.1% Triton X-100. Non-specific antigen was blocked by 10% Goat Serum. All the steps above were performed at room temperature. Primary E-cadherin antibodies (CST, 3195, RRID: AB_2291471) were incubated overnight at 4 °C and Isotype Normal Rabbit IgG (RD, AB-105-C) was utilized as a control. Secondary Goat anti Rabbit IgG antibody with Alexa Fluor 488 (Invitrogen, A11034, RRID: AB_2576217, Eugene, Oregon, USA) was incubated for 1 h at room temperature. F-actin was stained with Phalloidin FITC Reagent (Abcam, ab235137, Berlin, Germany) for 1 h. The nucleus was stained with DAPI (Thermo Scientific, V13069211, Eugene, OR, USA) for 10 min. Images were acquired and processed using an Evos FL Auto 2 imaging system (Invitrogen by Thermo Fisher Scientific, Bothell, WA, USA). Quantitative analyses of E-cadherin staining at cell–cell junctions were completed using imageJ.

### 2.12. Gene Set Enrichment Analysis (GSEA) and Gene Ontology (GO) Analysis

The expression profiles of pancreatic cancer cell lines were downloaded from CCLE through the DepMap Portal. The cell lines were ranked in decreasing order according to the median expression of PCSK6. The three cell lines ranked at the top (SUIT-2, SU8686 and ASPC1) and three cell lines at the bottom (SW1990, HS766T and CFPAC1) were assigned to PCSK6-high group and PCSK6-low group, respectively. In total, 16,383 genes in PCSK6-high group and PCSK6-low group were included in GSEA. Hallmark gene sets from the Molecular Signatures Database (MSigDB v7.4 www.gsea-msigdb.org/gsea/msigdb, accessed on 15 September 2021) were used to perform GSEA (GSEA v4.2.3). NES was generated and normalized *p*-values of the gene set <0.05 were considered statistically significant.

The R package clusterProfilter was adopted to perform GO analysis. Candidate pathways generated by GSEA were sorted into biological process (BP), molecular function (MF) and cell component (CC) categories. A network plot was established based on the target BP categories to provide an overview of the relationships among these categories and to identify core genes.

Potential proteolysis sites or motifs within target proteins were analyzed via the ELM database (the Eukaryotic Linear Motif resource for Functional Sites in Proteins), which provide a repository of annotated motif data and is a valuable tool for motif prediction (http://elm.eu.org/, accessed on 25 September 2021).

### 2.13. Western Blot

The total protein was lysed using the Pierce^TM^ RIPA Buffer (Thermo Scientific, 89900, Rockford, IL, USA) mixed with Halt^TM^ Protease and Phosphatase Inhibitor Cocktail (Thermo Scientific, 78442, Rockford, IL, USA). The quantitation of proteins was carried out using a Pierce^TM^ BCA Protein Assay Kit (Thermo scientific, 23227, Rockford, IL, USA). Protein samples were prepared by mixing an equal amount of protein with LDS Sample Buffer (Novex^TM^ by life technologies, B0008, Carlsbad, CA, USA) and Sample Reducing Agent (Novex^TM^ by life technologies, B0009) before denaturation. Gel electrophoresis was performed using a Mini Gel Tank chamber system (Invitrogen) with Bolt^TM^ 4–12% Bis-Tris gel (Invitrogen, NW04122BOX, Carlsbad, CA, USA), followed by transferring the protein to the Nitrocellulose Blotting membrane (Amersham^TM^, 10600003, Germany). 5% BSA or nonfat milk was used to block nonspecific antigens, and primary antibodies were incubated overnight at 4 °C, including phospho-p44/42 ERK1/2 (CST, 4370, RRID: AB_2315112), p44/42 ERK (CST, 4695, RRID: AB_390779) and GAPDH (CST, 5174, RRID: AB_10622025). HRP-linked Anti Rabbit IgG (CST, 7074, RRID: AB_2099233) was used as secondary antibody. Examination of the signal was performed using Amersham Imager 600 (Pittsburgh, PA, USA) with SignalFire™ ECL Reagent (CST, 6883S, Frankfurt, Germany).

### 2.14. Tumor Xenograft Model of Intrasplenic Injection

Intrasplenic injection was performed to establish liver metastasis. Briefly, 6-week-old NSG mice (NOD.Cg-Prkdc^scid^/l2rg^tm1Wjl^/Szj, The Jackson Laboratory, RRID: IMSR_JAX: 005557) were prepared via inhalation anesthesia with 2% Isoflurane, and intraperitoneal analgesia with 1% ketamine chloride and 0.1% xylazine chloride. A left flank incision was made to expose and move the spleen out of the abdominal cavity. Then, 4 × 10^4^ tumor cells resuspended in 20 μL PBS were injected into the lateral end of the spleen with a 30 G insulin syringe, followed by pressing the injection site with a cotton swab for 1 min to prevent leakage and facilitate hemostasis. Splenic vessels and gastrosplenic ligament were ligated 5 min after injection, and the spleen was removed to avoid tumor formation in the spleen. The abdominal wall was closed with a 5-0 suture (ETHICON, 1666H, San Lorenzo, Puerto Rico, USA). Three weeks after establishing the model, mice were sacrificed, and an exploration was carried out to rule out the planting lesions of tumors within the abdominal cavity. The whole liver was resected for pathological analysis, including macroscopic examinations and microscopic investigations of histological sections with hematoxylin and eosin (HE) staining. Four NSG mice were applied in each group (SUIT-SC and SUIT-NC). Three cross-sections of each liver sample were subjected to HE staining, and each section was scanned and transformed into a digital image. A total of 12 digital images for each group were utilized to calculate the counts of liver metastatic niches. The sizes of metastatic niches were determined using NDP.view 2.7.39 software. All the animal experiments were approved by the governments of Mittelfranken or Unterfranken, Germany. All the procedures mentioned above, animal housing, and animal husbandry were performed in accordance with the ethical regulations of the governments of Mittelfranken or Unterfranken, Germany.

### 2.15. Statistical Analysis

The statistical methods used in bioinformatics analysis have been mentioned above. Moreover, the variables in this study were continuous variables and are presented as means ± standard deviation (SD). The two-tailed unpaired Student’s t-test was utilized for comparisons between two groups. One-way ANOVA was utilized for comparisons among three or more groups, followed by multiple comparisons between groups using the Scheffe method. *p*-values < 0.05 were considered statistically significant. Different levels of *p*-values were defined as * <0.05, ** <0.01 and *** <0.001. All the statistical analyses were performed using the SPSS software 26.0 (SPSS Inc, Chicago, IL, USA).

## 3. Results

### 3.1. Identification of PCSK6 as a Liver-Metastasis-Related Gene

One hundred and eighty-one data series were collected from GEO, among which eight data series included samples of primary tumor and adjacent normal tissue, and two data series (GSE42952 and GSE71729) included samples of both liver metastasis and primary tumor (Table S2).

**Figure 1:**
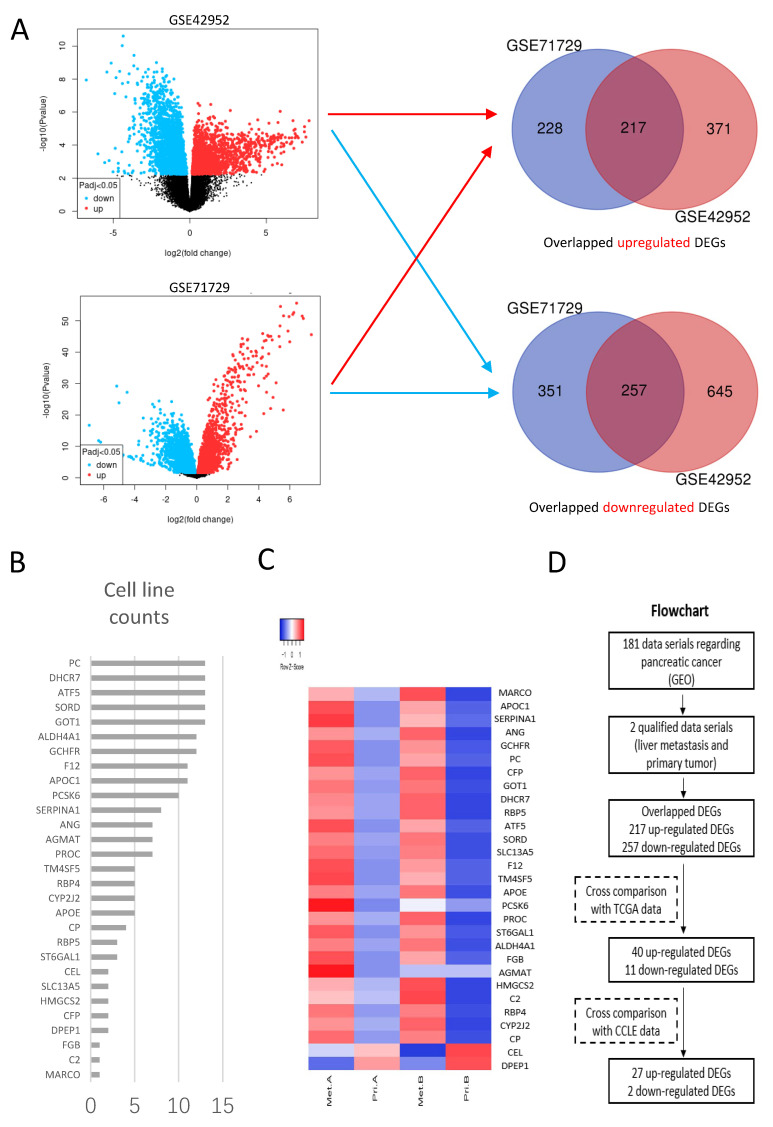
Bioinformatics analysis used to identify liver-metastasis-related genes. (**A**) Volcano plots of DEGs generated from data serials GSE42952 and GSE71729. Overlapping DEGs of up-regulated and down-regulated DEGs obtained via Venn diagrams. (**B**) Expressions of 29 candidate DEGs in cell lines of pancreatic cancer. (**C**) Heatmap of 29 candidate DEGs. Met A and Pri A represent liver metastasis and primary tumors of GSE71729. Met B and Pri B represent liver metastasis and primary tumors of GSE42952. (**D**) Flowchart of bioinformatics analysis.

Data series GSE42952 and GSE71729 were analyzed through GEO2R, and volcano plots of DEGs in each data series were shown in Figure 1A. One thousand four hundred and ninety DEGs were identified between liver metastasis and primary tumors in GSE42952, including 588 up-regulated and 902 down-regulated DEGs. One thousand and fifty-three DEGs were identified between liver metastasis and primary tumors in GSE71729, including 445 up-regulated and 608 down-regulated DEGs. The overlapping DEGs between the two data series consisted of 217 up-regulated DEGs and 257 down-regulated DEGs, and these were designated as liver-metastasis-related DEGs (L-DEGs).

Data from TCGA and GTEx were used to generate DEGs between primary tumors of pancreatic cancer and adjacent normal tissue, and these were designated as primary-tumor-related DEGs (P-DEGs). Through a comparison between L-DEGs and P-DEGs, 40 out of 217 up-regulated L-DEGs were found in up-regulated P-DEGs, and 11 out of 257 down-regulated L-DEGs were found in down-regulated P-DEGs. In the following step, through a verification with the expression profiles of cell lines (Table S3), 27 out of 40 up-regulated L-DEGs and 2 out of 11 down-regulated L-DEGs identified in the previous step were confirmed (Figure 1B). The final generated L-DEGs were represented using a heat plot in Figure 1C. A flowchart of the bioinformatics analysis process was also presented (Figure 1D).

**Figure 2:**
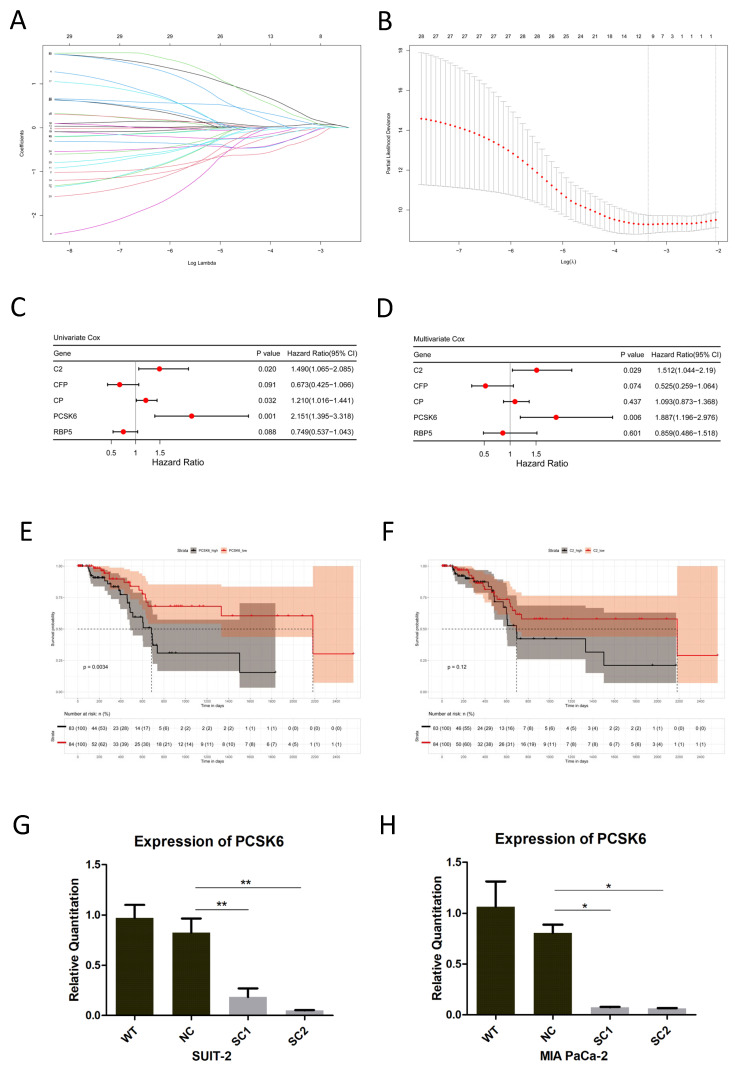
Prognostic analysis of candidate genes and knockout of PCSK6 in the cell lines SUIT-2 and MIA PaCa-2. (**A**) Lasso Cox regression analysis of 29 liver metastasis related genes. *y*-axis represents lasso coefficients and *x*-axis represents -log (Lambda) values. (**B**) Cross validation of the obtained genes via Lasso Cox regression. *y*-axis represents partial likelihood deviance and *x*-axis represents -log (Lambda) values. Dotted vertical line indicates the minimum and 1 standard error of Lambda values. The minimum values of Lambda were used as punishment coefficients for analysis. (**C**,**D**) Forest plots of univariate and multivariate Cox analysis. Five genes (*p* < 0.1) obtained via univariate analysis were further included in the multivariate analysis. (**E**,**F**) Kaplan–Meier survival plots of PCSK6 and C2. (**G**) Expression of PCSK6 mRNAs in SUIT-SCs and controls. (**H**) Expression of PCSK6 mRNAs in MIA-SCs and controls.

Among the 29 candidate genes, Lasso Cox regression identified 10 prognostic genes (Figure 2A,B and Table S4), which were further analyzed via univariate Cox regression. Five prognostic genes including C2, CFP, CP, PCSK6 and RBP5 (*p* < 0.1) identified in univariate analysis were included in the multivariate Cox regression analysis (Figure 2C,D), whereas only PCSK6 and C2 were independent prognostic genes. Survival plots of the prognostic genes PCSK6 and C2 obtained in the multivariate analysis were shown in Figure 2E,F. PCSK6 was closely correlated to patients’ survivals in pancreatic cancer (*p* = 0.0034), however, C2 was not statistically significant (*p* = 0.12). Therefore, we chose PCSK6 for further validations.

### 3.2. Knockout of PCSK6 in Cell Lines of Pancreatic Cancer

As PCSK6 was up-regulated in pancreatic cancer, inactivation of PCSK6 in both SUIT2 and MIA PaCa-2 was investigated in this study. Two sgRNAs targeting different regions in exons of PCSK6 (NC_000015.10, 100,166–100,188 bp and 105,294–105,316 bp) were designed, and CRISPR/Cas9 Gene Editing was performed to introduce mutations into the regions mentioned above. The transcription of PCSK6 mRNAs was inhibited, and these were verified via RT-PCR (Figure 2G,H). The expression of PCSK6 mRNAs in single clones of SUIT-2 was significantly down-regulated compared to controls (SC1 0.1840 ± 0.0860, SC2 0.0512 ± 0.0039, WT 0.9707 ± 0.2592, NC 0.8260 ± 0.2786, WT vs. NC *p* = 0.779, NC vs. SC1 *p* = 0.005, and NC vs. SC2 *p* = 0.001). The expression of PCSK6 mRNA in single clones of MIA PaCa-2 was significantly down-regulated compared to controls (SC1 0.0747 ± 0.0057, SC2 0.0649 ± 0.0025, WT 1.0638 ± 0.4955, NC 0.8062 ± 0.1636, WT vs. NC *p* = 0.598, NC vs. SC1 *p* = 0.015, and NC vs. SC2 *p* = 0.014). The mutations of genomic DNA in single clones of both SUIT-2 and MIA PaCa-2 were confirmed via DNA sequencing (Figure S1A–D). Western blot was not applied to determine the protein level of PCSK6 because of the most frequently cited anti-PACE4 antibody (Abcam, EPR8320, ab151562, Cambridge, UK) was not suitable for validating the knockout of PCSK6 (according to manufacturer’s notice).

### 3.3. Knockout of PCSK6 Suppressed Cell Proliferation

**Figure 3:**
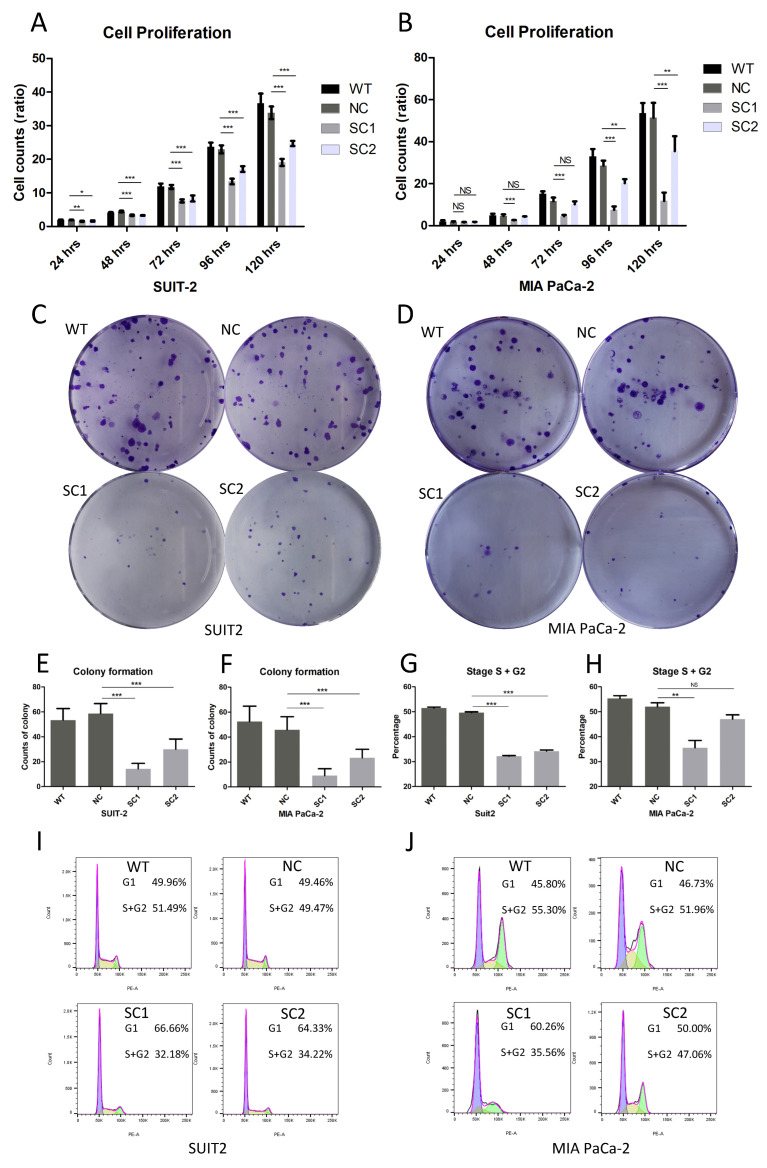
Knockout of PCSK6 suppressed cell proliferation. (**A**) Cell proliferation ratios of SUIT-SCs and controls at 24 h, 48 h, 72 h, 96 h and 120 h were analyzed, respectively (WT vs. NC *p* = 0.997, NC vs. SC1 *p* = 0.004 and NC vs. SC2 *p* = 0.023 at 24 h. WT vs. NC *p* = 0.118, NC vs. SC1 *p* < 0.001 and NC vs. SC2 *p* < 0.001 at 48 h. WT vs. NC *p* = 0.994, NC vs. SC1 *p* < 0.001 and NC vs. SC2 *p* < 0.001 at 72 h. WT vs. NC *p* = 0.775, NC vs. SC1 *p* < 0.001 and NC vs. SC2 *p* < 0.001 at 96 h. WT vs. NC *p* = 0.171, NC vs. SC1 *p* < 0.001 and NC vs. SC2 *p* < 0.001 at 120 h). (**B**) Cell proliferation ratios of MIA-SCs and controls at 24 h, 48 h, 72 h, 96 h and 120 h were analyzed, respectively (WT vs. NC *p* = 0.540, NC vs. SC1 *p* = 0.981 and NC vs. SC2 *p* = 0.999 at 24 h. WT vs. NC *p* = 0.890, NC vs. SC1 *p* < 0.001 and NC vs. SC2 *p* = 0.692 at 48 h. WT vs. NC *p* = 0.006, NC vs. SC1 *p* < 0.001 and NC vs. SC2 *p* = 0.427 at 72 h. WT vs. NC *p* = 0.079, NC vs. SC1 *p* < 0.001 and NC vs. SC2 *p* = 0.001 at 96 h. WT vs. NC *p* = 0.954, NC vs. SC1 *p* < 0.001 and NC vs. SC2 *p* = 0.006 at 120 h). (**C**,**E**) Colony formation of SUIT-SCs and controls. (**D**,**F**) Colony formation of MIA-SCs and controls. (**G**) Stage S + G2 of SUIT-SCs and controls. (**H**) Stage S + G2 of MIA-SCs and controls. (**I**) Cell cycle histogram of SUIT-SCs and controls. (**J**) Cell cycle histogram of MIA-SCs and controls.

A significant decrease in the proliferation ratio was found in SUIT-SC1 and SUIT-SC2 compared to controls via the proliferation assay (Figure 3A). The proliferation ratios of single clones of MIA PaCa-2 were inhibited after 48 h compared to controls via the proliferation assay (Figure 3B).

Significantly decreased counts of colony formation were observed in single clones of SUIT-2 compared to controls (SC1 14.3333 ± 4.3011, SC2 30.0000 ± 8.2613, WT 53.4444 ± 9.2751, NC 58.5556 ± 8.0794, WT vs. NC *p* = 0.584, NC vs. SC1 *p* < 0.001, and NC vs. SC2 *p* < 0.001), and single clones of MIA PaCa-2 compared to controls (SC1 9.1111 ± 5.5777, SC2 23.4444 ± 6.7659, WT 52.4444 ± 12.4107, NC 45.8889 ± 10.4096, WT vs. NC *p* = 0.525, NC vs. SC1 *p* < 0.001, and NC vs. SC2 *p* < 0.001). Additionally, the sizes of colonies were obviously smaller in single clones of both SUIT-2 and MIA PaCa-2 compared to their respective controls (Figure 3C–F).

Stages S and G2 of the cell cycle indicated the status of cell division. The proportions of stage S + G2 cells in single clones of SUIT-2 were diminished compared to controls (Figure 3G,I) (SC1 32.1833 ± 0.4900, SC2 34.2267 ± 0.7184, WT 51.4967 ± 0.6901, NC 49.4700 ± 0.8126, WT vs. NC *p* = 0.043, NC vs. SC1 *p* < 0.001, and NC vs. SC2 *p* < 0.001). A lower proportion of stage S + G2 cells was also observed in one single clone of MIA PaCa-2 compared to controls (Figure 3H,J) (SC1 35.5667 ± 5.1081, SC2 47.0667 ± 2.8936, WT 55.3000 ± 1.9975, NC 51.9667 ± 2.8676, WT vs. NC *p* = 0.708, NC vs. SC1 *p* = 0.003, and NC vs. SC2 *p* = 0.430).

### 3.4. Knockout of PCSK6 Inhibited Cell Migration

**Figure 4:**
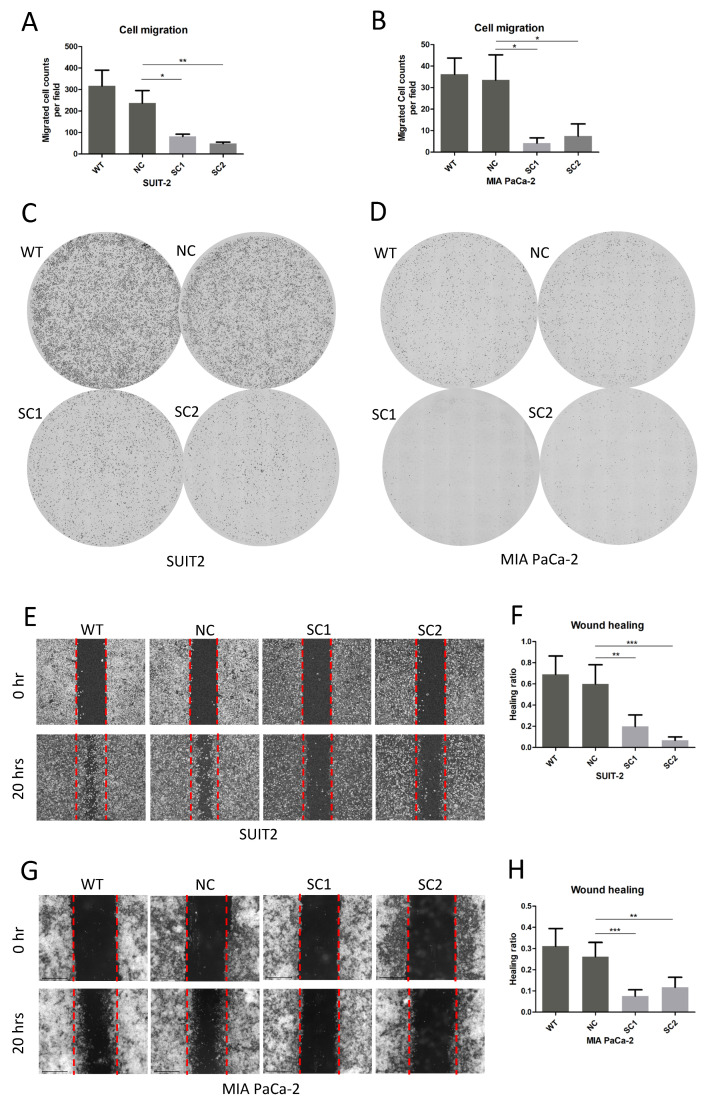
Knockout of PCSK6 inhibited cell migration. (**A**,**C**) Migration assay and counts of migrated cells in SUIT-SCs and controls. (**B**,**D**) Migration assay and counts of migrated cells in MIA-SCs and controls. (**E**,**F**) Wound-healing assay results and healing ratio in SUIT-SCs and controls. (**G**,**H**) Wound-healing assay results and healing ratio in MIA-SCs and controls.

In the migration assay, fewer cells migrated to the inferior surface of the superior chamber in single clones of SUIT-2 compared to controls within 20 h (Figure 4A,C) (SC1 81.9222 ± 10.4127, SC2 48.6778 ± 6.6381, WT 316.7111 ± 73.5682, NC 236.9444 ± 58.0643, WT vs. NC *p* = 0.306, NC vs. SC1 *p* = 0.025, and NC vs. SC2 *p* = 0.009). Similarly, single clones of MIA PaCa-2 migrated more slowly than controls within 20 h (Figure 4B,D) (SC1 4.2444 ± 2.3856, SC2 7.4889 ± 5.6151, WT 36.2000 ± 7.5684, NC 33.5111 ± 11.7365, WT vs. NC *p* = 0.978, NC vs. SC1 *p* = 0.011, and NC vs. SC2 *p* = 0.021).

In the wound healing assay, the healing ratios in single clones of SUIT-2 decreased significantly compared to controls (Figure 4E,F) (SC1 0.1984 ± 0.1089, SC2 0.0671 ± 0.0324, WT 0.6900 ± 0.1740, NC 0.5992 ± 0.1819, WT vs. NC *p* = 0.732, NC vs. SC1 *p* = 0.001, and NC vs. SC2 *p* < 0.001). Consistent results were found in single clones of MIA PaCa-2 compared to controls (Figure 4G,H) (SC1 0.0760 ± 0.0294, SC2 0.1177 ± 0.0466, WT 0.3116 ± 0.0825, NC 0.2621 ± 0.0668, WT vs. NC *p* = 0.572, NC vs. SC1 *p* < 0.001, and NC vs. SC2 *p* = 0.005).

**Figure 5:**
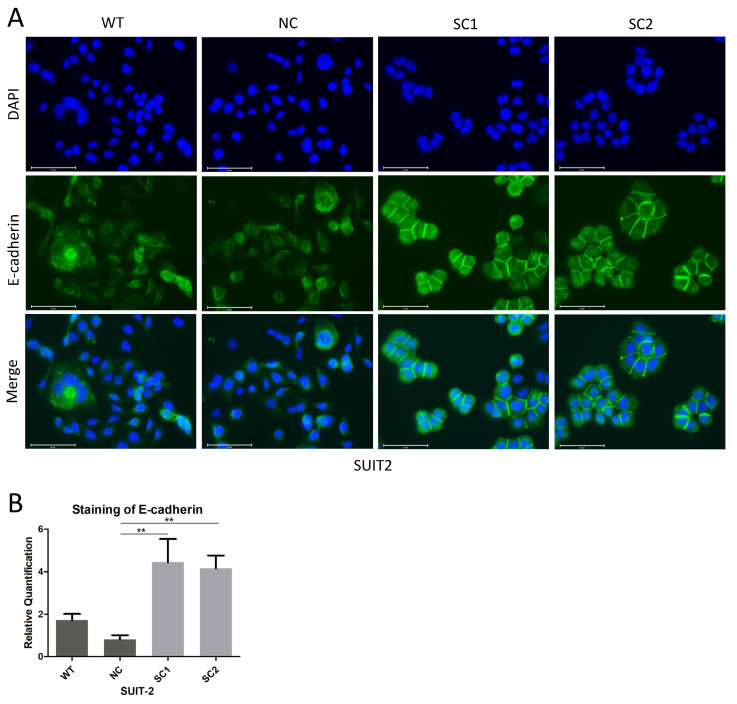
Knockout of PCSK6 induced the redistribution of E-cadherin at cell–cell junctions. (**A**) Immunofluorescence staining of E-cadherin (green), DAPI (blue), and merge in SUIT-SCs and controls. Scale bar = 75 μm. Diffuse and strong staining of E-cadherin was found at cell–cell gaps in single clones of SUIT-2, whereas scattered and weak staining of E-cadherin was found in WT or NC. (**B**) Relative quantification of E-cadherin staining at cell–cell junctions in SUIT-SCs and controls. SC1 = 4.4453 ± 1.0974, SC2 = 4.1570 ± 0.6033, WT = 1.7237 ± 0.2899, NC = 0.8093 ± 0.2007 (WT vs. NC *p* = 0.446, NC vs. SC1 *p* = 0.001, NC vs. SC2 *p* = 0.002).

**Figure 6:**
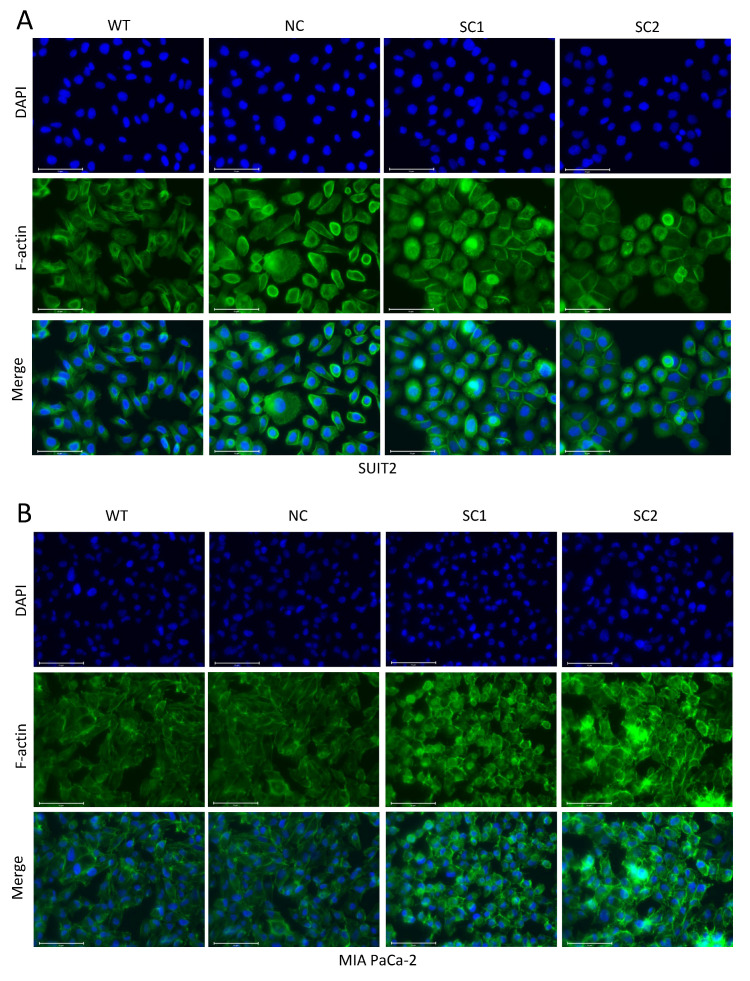
Knockout of PCSK6 induced remodeling of the cell-skeleton. (**A**) Immunofluorescence staining of F-actin (green), DAPI (blue), and merge in SUIT-SCs and controls. Scale bar = 75 μm. Single clones of SUIT-2 turned to be cubic or round in shape, with similar sizes, and formed epithelial-like clusters, whereas WT or NC remained in spindle or fusiform shapes, with significantly diverse sizes, and lost their polarities and lined up irregularly. (**B**) Immunofluorescence staining of F-actin (green), DAPI (blue), and merge in MIA-SCs and controls. Scale bar = 75 μm. Single clones of MIA PaCa-2 adopted a cubic shape and exhibited similar sizes, however, WT and NC remained spindle-shaped or fusiform with significantly diverse sizes.

To explore whether the decreased capacity of cell migration in single clones of either SUIT-2 or MIA PaCa-2 was related to variations in cell–cell junctions or the cell skeleton, the expression levels of E-cadherin and F-actin were investigated via immunofluorescence. Restoration of E-cadherin expression was observed at cell–cell gaps in SUIT-2 after knockout of PCSK6 (Figure 5A,B). Single clones and controls of MIA PaCa-2 showed no obvious staining of E-cadherin (Figure S2). Through the staining of F-actin, we found that both SUIT-2 and MIA-PaCA-2 changed their cell morphology, cell polarity, or lining style after the knockout of PCSK6 (Figure 6A,B).

### 3.5. Knockout of PCSK6 Decreased Counts and Outgrowth Rates of Metastatic Niches in Liver

**Figure 7:**
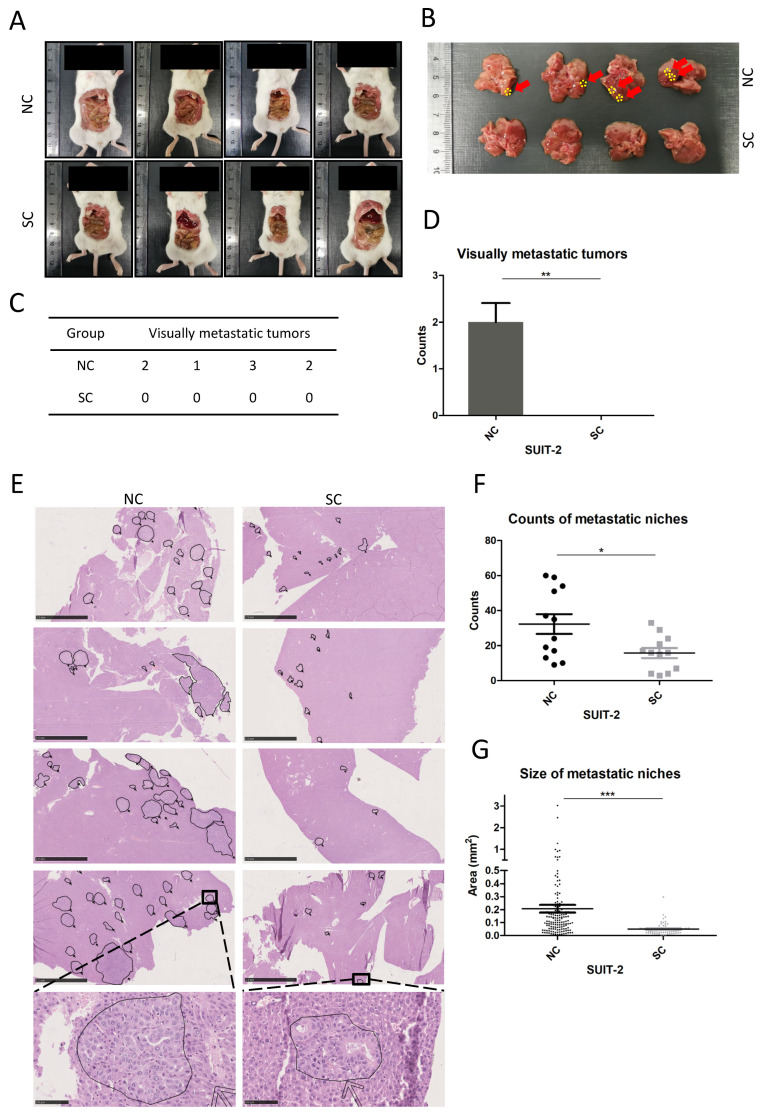
Knockout of PCSK6 inhibited liver metastasis in the tumor xenograft model of intrasplenic injection. (**A**) Overall explorations of the abdominal cavity in each group. (**B**–**D**) Macroscopic examinations of liver metastatic lesions in each group. (**E**) Microscopic view of HE sections, with identified liver metastatic niches in each group. Each metastatic niche was marked, and the area was calculated automatically. Scale bar = 2.5 mm. (**F**) Counts of liver metastatic niches in each group. (**G**) Sizes of liver metastatic niches in each group.

The tumor xenograft model of intrasplenic injection simulated the processes of tumor cells colonizing in the liver via the bloodstream, interacting with the microenvironment and generating metastatic niches. No tumor-planting lesions or ascites within the abdominal cavity were evident (Figure 7A). In macroscopic examinations (Figure 7B–D), prominent metastatic lesions were found at different lobes of the liver in the NC group of SUIT-2, in contrast no visually metastatic lesions were found in the SC group (NC 2 ± 0.408, SC 0, *p* = 0.003). In microscopic investigations, the SC group of SUIT-2 showed fewer counts of metastatic niches in the liver compared to the NC group (Figure 7F) (SC 15.75 ± 9.937, NC 32.33 ± 19.593, *p* = 0.019). Furthermore, obviously smaller sizes of metastatic niches within the liver were found in the SC group of SUIT-2 compared to the NC group (Figure 7E,G) (SC 0.0498 ± 0.0434, NC 0.2070 ± 0.3597, *p* < 0.001).

### 3.6. Knockout of PCSK6 Suppressed Raf-MEK1/2-ERK1/2 Axis

**Figure 8:**
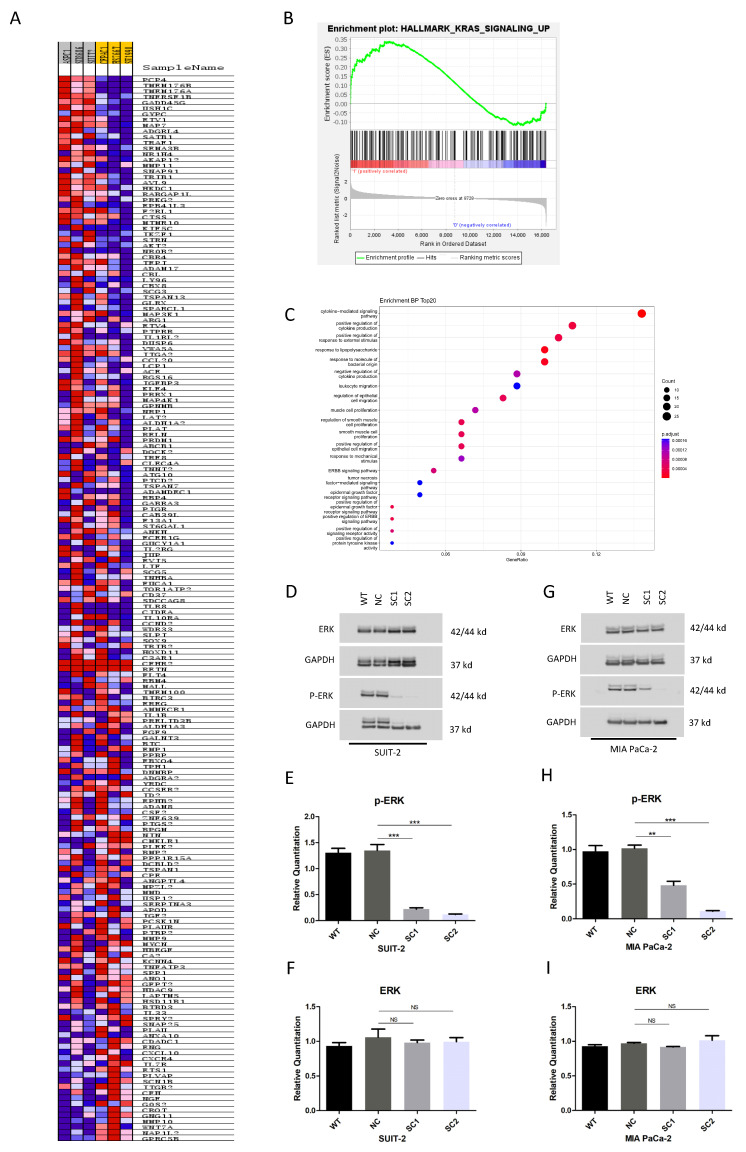
Knockout of PCSK6 suppressed the Raf-MEK1/2-ERK1/2 axis. (**A**) Heatmap of the expressions of genes involved in the Kras signaling pathway between the PCSK6-high group and the PCSK6-low group. (**B**) Enrichment plot of the Kras signaling pathway. (**C**) Top 20 enrichment categories of BP. (**D**–**F**) Activities of ERK and p-ERK in SUIT-SCs and controls. (**E**,**F**): relative quantitation of p-ERK and ERK in Western blot (Intensity ratios of the bands). (**G**–**I**) Activities of ERK and p-ERK in MIA-SCs and controls. (**H**,**I**): relative quantitation of p-ERK and ERK in Western blot (Intensity ratios of the bands). Additional bands at 42/44 kd locating superior to GAPDH bands resulted from reprobing of the membrane in (**D**,**G**).

The KRAS mutation was one of the most frequent oncogenic events in pancreatic cancer. Mutant KRAS resulted in the continuous activation of the Ras-Raf-MEK1/2-ERK1/2 signaling axis. Through GESA, we noted that the high expression of PCSK6 was correlated with the up-regulation of the Kras signaling pathway compared to the low expression of PCSK6 in the cell lines of pancreatic cancer (Figure 8A,B). Accordingly, high expression of PCSK6 may enhance the Kras signaling pathway, and its associated gene profile was included in the GO analysis. The top 20 enrichment categories of BP, MF and CC were listed in decreasing order (Figure 8C, Figure S3A,B). In the BP analysis, cell proliferation and migration-related enrichment categories, such as the regulation of epithelial cell migration, the epidermal growth factor receptor singling pathway, the regulation of protein tyrosine kinase activity, etc., were identified. Using a network plot of BP categories, we found that PCSK6-associated core genes included HBEGF, ADAM17, MMP9, SOX9, BTC and EREG, which may participate in cell growth or cell migration (Figure S3C).

One of the downstream signaling pathways of Kras was Raf-MEK1/2-ERK1/2 axis. Through linear motif analysis using the ELM database, we observed putative proteolysis sites of proprotein convertases among the linear amino sequence of Kras, RAF, MEK1/2 and ERK1/2 (Table S5).

After the knockout of PCSK6 was performed in SUIT-2 (Figure 8D–F), no obvious variation was found in the expression of ERK1/2 (SC1 0.9805 ± 0.0647, SC2 0.9942 ± 0.1048, WT 0.9327 ± 0.0828, NC 1.0600 ± 0.2033, WT vs. NC *p* = 0.686, NC vs. SC1 *p* = 0.894, and NC vs. SC2 *p* = 0.935), however, significant inhibitions of phosphorylated ERK1/2 were identified in both single clones compared to controls (SC1 0.2230 ± 0.0438, SC2 0.1200 ± 0.0172, WT 1.3057 ± 0.1463, NC 1.3490 ± 0.1979, WT vs. NC *p* = 0.980, NC vs. SC1 *p* < 0.001, and NC vs. SC2 *p* < 0.001). Similar results were found in MIA PaCa-2 (Figure 8G,I), that no obvious change was observed in the expression of ERK1/2 (SC1 0.9170 ± 0.0118, SC2 1.01482 ± 0.1130, WT 0.9285 ± 0.0354, NC 0.9708 ± 0.0206, WT vs. NC *p* = 0.863, NC vs. SC1 *p* = 0.759, and NC vs. SC2 *p* = 0.849), whereas significant repressions of phosphorylated ERK1/2 were identified (SC1 0.4803 ± 0.1055, SC2 0.1107 ± 0.0149, WT 0.9730 ± 0.1433, NC 1.0155 ± 0.0829, WT vs. NC *p* = 0.962, NC vs. SC1 *p* = 0.001, and NC vs. SC2 *p* < 0.001). The intensity ratios of the bands in Western blot were summarized in Tables S6 and S7. The original figures in Western blot were uploaded in Supplementary Materials.

## 4. Discussion

The TME is comprised of tumor cells and the mesenchyme, including cellular components and non-cellular components. The interactions between tumor cells and the mesenchyme were found to induce variations in molecular profiles of tumor cells and remodel the TME, finally promoting tumor progression [18]. The biological behaviors of tumor cells are governed by alterations of driver genes, however, epigenetic, post-transcriptional, and post-translational modifications coupled with the TME have emerged as equally critical determinants during the course of tumor development [19]. Therefore, the TME of liver metastatic niches may provide us with new clues to find genes related to the liver metastasis of pancreatic cancer. We carried out unique bioinformatics analysis of the mRNA expression profiles in both liver metastasis and primary tumors, and identified 29 candidate genes. One of the obtained genes, PCSK6, was significantly prognostic and this was chosen for further study.

PCSK6 locates at 15q26.3 and is the coding gene of PACE4, a member of the proprotein convertase family and a kind of calcium-dependent serine proteinase which participates in post-translational modifications [20]. The proprotein convertase family consists of nine currently known members, including PC1, PC2, furin, PC4, PC5, PACE4, PC7, SKI-1, and PCSK9. With the exception of SKI-1 and PCSK9, the remaining seven members of the proprotein convertase family have been found to activate various protein precursors through proteolysis at specific single or paired basic amino acids within the motif (R/K)Xn(R/K)↓, however, there are variations in cleavage sites for each proprotein convertase [14]. PACE4 is synthesized as an inactive precursor and then it is converted into a mature enzyme via both autoactivation in the endoplasmic reticulum and activation by other proteinases, which are prerequisites for processing its substrates [21]. Up-regulation of PCSK6 in the liver metastasis of pancreatic cancer was identified in this study. The detailed functions of PCSK6 in tumor cells and its impact on the liver metastasis of pancreatic cancer remain to be determined. CRISPR/Cas9, a cutting-edge gene editing technology, was utilized to inactivate PCSK6 in this study. Through the accurate binding of sgRNA guided Cas9 nuclease and genome DNA, exons of PCSK6 were edited at designated sites, which were proven via genome DNA sequencing. Variations in base sequences among exons resulted in the decreased efficiency of mRNA transcription and significant inhibitions of PCSK6 mRNA expression, which were confirmed via RT-PCR.

The results that single clones of both SUIT-2 and MIA PaCa-2 showed lower proliferation rates and fewer colony formation counts compared to controls, indicated that the inactivation of PCSK6 could inhibit the growth of tumor cells in pancreatic cancer. G0/G1 cell cycle arrest with decreased ratios and decreased trends in the S/G2 phase, were demonstrated in single clones of SUIT-2 and MIA PaCa-2, respectively, at least in part validating the above results. Similar findings were also obtained in skin cell tumors [15,22] and prostate cancer [23,24]. PACE4 acts through calcium-dependent serine proteases and shows a preference for the cleavage sites of (R/K)Xn(R/K), accordingly its substrates include various growth factors, metalloproteinases, etc. [14]. We postulated that the inactivation of PCSK6 attenuated the interactions between PACE4 and its substrates, leading to immature growth factors and decreased downstream signaling, mediating the growth of tumor cells.

In addition, the inactivation of PCSK6 in single clones of both cell lines could also suppress cell migration, according to the results of the migration assay and wound-healing assay in this study. Most previous researchers focused on the effect of PCSK6 on cell growth or apoptosis [23,24,25], whereas one study noted that the elevated expression of PCSK6 enhanced the migratory capacity of skin tumor cells [22], which was consistent with our findings in relation to pancreatic cancer. Molecular reprogramming in tumor cells may induce modifications of cell–cell junctions or the cell skeleton, which play a critical role in cell movement, deformation, etc. [26]. E-cadherin is a component of the stable adherens junctions connecting the actin skeletons of adjacent cells [27]. Proprotein convertases including PC7, furin, and PACE4 have been proven to regulate cell–cell junctions by processing E-cadherin during blastocyst formation [28]. Moreover, we found specific motifs or cleavage sites for proprotein convertases within the amino acids sequence of E-cadherin (Table S5). Therefore, we examined the expression of E-cadherin, and it was worth noting that, instead of decreasing the expression of E-cadherin, the inactivation of PCSK6 enhanced the expression of E-cadherin at sites of cell–cell junctions in single clones of SUIT-2, although not in MIA PaCa-2, which showed an extremely low expression of E-cadherin [29,30]. Previous studies have shown that, when one of the proprotein convertases is inactivated, other members can process immature E-cadherin efficiently [31]. Accordingly, a possible explanation for our observations was that the maturation of E-cadherin was not dependent on PACE4, and other proprotein convertases probably played a compensatory role in processing premature E-cadherin. Alternatively, other unknown mechanisms might be responsible for the increased expression of E-cadherin after the inactivation of PCSK6 in SUIT-2 cells. Repression of the Raf-MEK-ERK signaling pathway would lead to restoration of E-cadherin [32,33] and this might be one of the reasons for the up-regulation of E-cadherin observed in this study. Furthermore, F-actin, acting in coordination with adherens junctions to direct cell morphology and cell movement, was found to be up-regulated in single clones of both cell lines. Specifically, transformation of cell morphology from spindle-shaped to cubic or round, as well as regaining of a glandule-like structure and apicobasal polarity, were features highlighted after the inactivation of PCSK6. Variation in cell–cell junctions and the cell skeleton enable tumor cells to adapt to different phases of tumor progression and to various mesenchymal components in the TME [27]. In summary, the inactivation of PCSK6 led to the decreased migratory capacity of tumor cells in pancreatic cancer, and reprogramming of cell–cell junctions or the cell skeleton might be one of the causes for this effect.

The final step in the metastatic process is the generation of colonies in the liver, which requires tumor cells to overcome serial obstacles to outgrow into a macroscopic metastasis, including getting access to the liver through blood vessels, gaining a nutritional supply or growth stimuli from surrounding tissue, remodeling or establishing the TME, and escaping from immune elimination [19]. In this study, tumor xenograft model of intrasplenic injection was used to simulate the colonization of tumor cells in the liver. Prominent macroscopic metastatic lesions were found in the NC group of SUIT-2 but not in the SC group. Microscopic metastatic niches were found in both groups of SUIT-2, whereas fewer counts and smaller-sized metastatic niches were observed in the SC group compared to the NC group. Considering that PACE4 can process and activate growth stimuli and adhesion molecules in the extracellular matrix (ECM) in the manner of a secretory protein [14], the results showing that the inactivation of PCSK6 made tumor cells less competent to establish metastatic niches in the liver and finally retarded tumor growth in vivo, might in part be attributed to the presence of fewer extravasating cells from the blood wall, fewer anchored cells in liver tissue, or less active growth stimuli surrounding tumor niches. Small molecule inhibitors targeting PACE4 have been investigated in relation to arthritis disease [34], human skin tumors [15] and human prostate cancer [35]. Accordingly, our preclinical animal model will provide evidence and options for future evaluations of small-molecule inhibitors of PACE4 or their combined use with chemotherapy in the treatment of liver metastasis in pancreatic cancer.

KRAS, CDKN2A, TP53 and SMAD4 are the most frequently observed driver genes involved with genetic mutations in pancreatic cancer, and KRAS mutations occur in 90% of the patients [36]. Through GSEA, we found that the high expression of PCSK6 was accompanied by the up-regulation of Kras signaling pathway compared to the group with a relatively low expression of PCSK6, in the cell lines of pancreatic cancer. The Raf-MEK1/2-ERK1/2 axis is identified as one of the downstream signaling pathways of Kras. Previously studies have linked furin, another member of the proprotein convertase family, to the activation of the Raf-MEK1/2-ERK1/2 pathway via endoproteolytic cleavage in KRAS or BRAF mutant cells [37]. As a ubiquitously distributed proprotein convertase in human tissues, PACE localizes subcellularly to the trans-Golgi network or the cell surface, and its substrates include hormones, tyrosine phosphatases, growth factors, metalloproteinases, and adhesion molecules in the extracellular matrix (ECM) or on the cell surface [38]. One possible mechanism is that PACE4-processing growth factors or other substrates on the cell surface or in the ECM might exert an effect on the Raf-MEK1/2-ERK1/2 axis. We evaluated the impact of PCSK6 inactivation on the Raf-MEK1/2-ERK1/2 axis in this study. Strikingly, compared to controls, single clones of both cell lines showed extremely low activities of phosphorylated ERK1/2 in this study, which is a critical modulator regulating the transcriptions of multiple genes correlated with the cell cycle, apoptosis, cell migration, cell invasion, etc. [39]. Since SUIT-2 and MIA PaCa-2 in this study are both cell lines with KRAS mutations and they activate the downstream Raf-MEK1/2-ERK1/2 axis, regardless of whether the upstream components such as RTK are muted due to immature ligands, there may be another reasonable explanation for the notable suppression of the Raf-MEK1/2-ERK1/2 axis after PCSK6 inactivation. Therefore, another potential mechanism is that Kras, Raf, MEK1/2, and ERK1/2 are potential substrates of proprotein convertases according to our analysis based on the predictions of the ELM database, which provide theoretical evidence for interactions between PACE4 and Kras or the Raf-MEK1/2-ERK1/2 axis, however, the current literature does not provide sufficient evidence for the interaction of PACE4 with Kras or the Raf-MEK1/2-ERK1/2 axis intracellularly [39,40]. More investigations will be necessary to confirm this.

Previous researchers found that PCSK6 regulated cell apoptosis via endoplasmic reticulum stress and the mitochondria signaling pathway [25]. We have advanced the knowledge by demonstrating that the inactivation of PCSK6 can significantly inhibit the Raf-MEK1/2-ERK1/2 axis, having a repressive effect on cell growth, cell migration and the generation of metastatic niches in the liver in pancreatic cancer. The epidermal growth factor receptor (EGFR) tyrosine kinase inhibitor has demonstrated limited survival benefits for patients with pancreatic cancer due to mutated KRAS [41], however, the inactivation of PCSK6 may be a novel target of treatment, bypassing mutated KRAS and retarding tumor progression in pancreatic cancer, providing that a direct interaction between PACE4 and the Raf-MEK1/2-ERK1/2 axis can be proven in the future.

## 5. Conclusions

We demonstrated here that the inactivation of PCSK6 in cell lines of human pancreatic cancer could inhibit cell growth via inducing G0/G1 cell cycle arrest, and could impede cell migration that relied on the remodeling of cell–cell junctions and the cell skeleton. The loss of PCSK6 in vivo repressed the generation of metastatic niches in the liver, decreasing both the counts and outgrowth rates of metastatic niches. We have updated the scientific knowledge in this area by demonstrating that the inactivation of PCSK6 could suppress the Raf-MEK1/2-ERK1/2 signaling pathway to restrain tumor progression and liver metastasis, thus providing a promising and attractive target for tailored therapies to overcome challenges from mutated KRAS, however, in-depth evaluations of this novel target and therapeutic strategy in pancreatic cancer remain essential.

## Data Availability

The data presented in this study are available in the article and Supplementary Materials.

## Supplementary Materials

The following data were available at https://www.mdpi.com/article/10.3390/cancers15010241/s1. Figure S1: Genomic DNA sequencing of single clones, Figure S2: E-cadherin in MIA PaCa-2, Figure S3: GO analysis of PCSK6 associated genes involved in the Kras signaling pathway, Table S1: Oligos for sgRNAs, PCR and DNA sequencing, Table S2: Characteristics of data series, Table S3: Expressing profiles of 51 L-DEGs in cell lines of pancreatic cancer, Table S4: Prognostic genes obtained by Lasso Cox regression, Table S5: Cleavage sites of proprotein convertases on target proteins from ELM database, Tables S6 and S7: The intensity ratios of the bands in Western blot, and the original figures in Western blot.
