# Supplementary material for: Identification of Genes Associated with Liver Metastasis in Pancreatic Cancer Reveals PCSK6 as a Crucial Mediator
Source: Cancers (Basel). 2022 Dec 30;15(1):241. doi: 10.3390/cancers15010241 (PMC9818395; doi:10.3390/cancers15010241)
Supplement: Supplementary file 1 [file cancers-15-00241-s001.zip › Table S1.pdf]

Supplemented Table S1 Oligos for sgRNAs, PCR and DNA sequencing

Names	Sequence (5’->3’)
Hu_PCSK6_sgRNA1_f	CACCGCTGTACCGATGGCCACACT
Hu_PCSK6_sgRNA1_r	AAACAGTGTGGCCATCGGTACAGC
Hu_PCSK6_sgRNA2_f	CACCGCAGGTGCTGGACGTCCCTCC
Hu_PCSK6_sgRNA2_r	AAACGGAGGGACGTCCAGCACCTGC
Hu_NC_sgRNA_f	CACCgCGGGACGTCGCGAAAATGTA
Hu_NC_sgRNA_r	AAACTACATTTTCGCGACGTCCCGC
Hu_PCSK6_f	GAGCGAAAAATCGTCACCAC
Hu_PCSK6_r	TCCAGTCGCTCGCTTTCAG
Hu_beta_actin_f	CACCATTGGCAATGAGCGGTTC
Hu_beta_actin_r	AGGTCTTTGCGGATGTCCACGT
Hu_DNA-sgRNA1_f	GCCTAACACCTTATTTGTTCCCGAG
Hu_DNA-sgRNA1_r	GGTGCACGACTCACTGAATGTGCTT
Hu_ DNA-sgRNA2_f	CAAGGAATGCAGTCCTTAGCCTGAG
Hu_ DNA-sgRNA2_r	TGCGTTTTAAATTCTTGTGACACAC
