# Supplementary material for: Identification of Genes Associated with Liver Metastasis in Pancreatic Cancer Reveals PCSK6 as a Crucial Mediator
Source: Cancers (Basel). 2022 Dec 30;15(1):241. doi: 10.3390/cancers15010241 (PMC9818395; doi:10.3390/cancers15010241)
Supplement: Supplementary file 1 [file cancers-15-00241-s001.zip › Table S2.pdf]

Supplemented Table S2 Characteristics of data serials

GSE accession	Platform	Primary tumor	Adjacent normal tissue	Liver metastasis	Author and year	Region
GSE62452	GPL6244	69	61		Yang et al. 2016	USA and Germany
GSE15471	GPL570	39	39		Badea et al. 2009	Romania
GSE62165	GPL13667	118	13		Janky et al, 2016	Belgium
GSE102238	GPL19072	50	50		Yang et al. 2017	China
GSE32688	GPL570	25	7		Donahue et al. 2011	USA
GSE43288	GPL96	4	3		Crnogorac et al. 2013	UK
GSE63111	GPL5188	28	6		Wang et al. 2017	UK
GSE23397	GPL5188	15	6		Gerhardinger et al. 2013	Germany
GSE42952	GPL570	12		7	Van et al. 2013	Belgium
GSE71729	GPL20769	145		25	Moffitt et al. 2015	USA
