# Supplementary material for: Identification of Genes Associated with Liver Metastasis in Pancreatic Cancer Reveals PCSK6 as a Crucial Mediator
Source: Cancers (Basel). 2022 Dec 30;15(1):241. doi: 10.3390/cancers15010241 (PMC9818395; doi:10.3390/cancers15010241)
Supplement: Supplementary file 1 [file cancers-15-00241-s001.zip › Table S3.pdf]

Supplemented Table S3 Expressing profiles of 51 L-DEGs in cell lines of pancreatic cancer

Gene	Cell line						
	ASPC1 ACH-000222	BXPC3 ACH-000535	CAPAN1 ACH-000354	CAPAN2 ACH-000107	CFPAC1 ACH-000138	HPAC ACH-270	HPAFII ACH-000094
ABCB4	0.111031	0.042644	0.189034	0.028569	0.584963	0.056584	0.163499
DPEP1	2.914565	0.464668	0.097611	0.367371	0.545968	0.014355	1.570463
MARCO	0.028569	2.31034	0.163499	0.070389	0.084064	0.111031	0.137504
HSD17B6	0.298658	0.62293	1.464668	0.31034	0.565597	0.389567	0.189034
CP	0.516015	1.31034	2.498251	0.400538	4.911212	4.120186	0.250962
LMO3	0.695994	0.250962	0.887525	0.722466	0.15056	1.405992	0.201634
ST6GAL1	0.124328	1.618239	3.343408	1.298658	1.799087	0.632268	0.176323
AQP9	0	0	0.014355	0.014355	0	0.028569	0
REG1A	0	0	0.238787	0.238787	0	0	0.056584
PROC	5.450222	1.411426	2.173127	2.488001	2.709291	1.641546	2.370164
AGMAT	3.664483	2.89724	2.568032	1.726831	0.704872	1.250962	3.163499
GOT1	4.965323	5.888743	4.877253	5.454834	5.138323	4.767655	4.864434
F10	0.286881	0.042644	0.782409	0.111031	0.189034	0.214125	0.263034
CFP	0.495695	0.356144	0.632268	0.604071	1.367371	0.367371	1.056584
APOE	0.475085	0.895303	2.987321	1.739848	3.304511	2.114367	0.669027
APOC1	3.301588	3.295723	4.245648	4.929791	3.534809	3.861955	1.035624
F12	4.832384	4.001802	4.67638	3.381283	3.889474	1.599318	4.999549
CRP	0	0	0.028569	0	0	0.014355	0
HMGCS2	0	0.042644	2.729009	0.084064	0.084064	0.137504	0.15056
CYP2J2	0.137504	2.456806	2.017922	1.275007	2.62527	1.70044	2.82985
GCHFR	6.708877	5.182692	3.104337	5.086614	6.197315	3.58376	4.475733
CYP2C9	0.056584	0.15056	0.485427	0.028569	0.411426	0.014355	0.056584
RBP4	4.056584	0.163499	1.599318	0.31034	2.367371	1.560715	4.849999
RBP5	1.996389	1.15056	1.726831	1.952334	1.104337	2.07382	2.140779
SORD	4.368768	6.474274	6.386294	4.392317	4.295723	4.339137	4.892391

Supplemented Table S3 continued

Gene	ASPC1 ACH-000222	BXPC3 ACH-000535	CAPAN1 ACH-000354	CAPAN2 ACH-000107	CFPAC1 ACH-000138	HPAC ACH-270	HPAFII ACH-000094
PCSK6	5.010332	4.926948	4.716442	3.791814	1.847997	3.731183	3.596935
SLC13A5	0	0.056584	0.201634	1.063503	2.518535	0.028569	1.941106
ASGR1	0.31034	0.594549	0.948601	0.070389	0.695994	1.035624	0.678072
TM4SF5	5.244507	0.097611	2.641546	2.361768	1.495695	0.056584	5.484783
ITIH2	0	0	0.014355	0	0.176323	0	0.042644
TDO2	0.042644	0.070389	0.070389	0.056584	0.782409	0.014355	0.014355
CPB1	0	0	0	0	0.070389	0	0.014355
ALDH4A1	1.510962	4.82171	3.139142	2.641546	2.613532	2.693766	2.757023
ITIH3	0	0.084064	0	0.084064	0.028569	0	0
C2	0.422233	1.584963	1.207893	0.263034	0.918386	0.097611	0.575312
CTRB2	0.097611	0.042644	0.333424	0.31034	0.485427	0.111031	0.176323
ATF5	4.412782	6.171927	4.395063	4.8166	4.014355	4.127633	5.013016
GP2	0	0	0.014355	0	0	0	0.014355
CEL	0.400538	2.443607	0.831877	0.613532	0.400538	1.056584	0.298658
FGG	0.014355	0.137504	0.137504	0	1.124328	0	0.137504
FGA	0	0.014355	0.028569	0.028569	1.189034	0	0
FGB	0	0.084064	0.111031	0.070389	3.132577	0.124328	0.189034
REG3A	0	0	0	0	0	0	0
REG1B	0	0	0	0	0	0	0
DHCR7	5.255501	6.54782	6.312701	8.147612	5.427271	5.372952	6.393348
PC	3.237258	4.334139	3.602884	3.454176	3.613532	2.650765	3.933573
PNLIP	0	0	0	0	0.014355	0	0
SERPINA1	0.815575	0.584963	4.390943	8.436212	7.755021	2.298658	6.59902
ANG	3.57289	1.049631	3.596935	2.223423	2.565597	1.589764	4.039138
APOC2	0.250962	0.163499	0	0.084064	0	0.344829	0.176323

Supplemented Table S3 continued

Gene	HS766T<br>ACH-000178	MIAPACA2<br>ACH-000601	PANC1<br>ACH-000164	SU8686<br>ACH-000114	SUIT2<br>ACH-000652	SW1990<br>ACH-000155
ABCB4	0.028569	0	0	1.521051	0.15056	0
DPEP1	0	0.084064	0.238787	0.137504	5.014802	0.15056
MARCO	0	0	0	0.389567	0	0.014355
HSD17B6	0.226509	0.799087	0.970854	1.090853	0.555816	0.238787
CP	0.565597	0.298658	0.773996	3.030336	1.41684	0.238787
LMO3	0.028569	0.056584	0.201634	0.83996	1.803227	0.111031
ST6GAL1	2.235727	1.819668	0.831877	4.331992	1.144046	0.137504
AQP9	0	0	0	0.042644	0	0
REG1A	0	0	0	0	0	0
PROC	0.613532	0.042644	2.107688	2.523562	1.594549	0.505891
AGMAT	2.275007	0.070389	2.01078	0.895303	4.074677	1.02148
GOT1	5.286142	5.20085	5.950702	5.910972	5.114367	4.924575
F10	0.028569	0.163499	0.275007	1.678072	1.895303	0.411426
CFP	0.321928	0.863939	0.298658	2.07382	0.097611	2.568032
APOE	0.933573	1.280956	7.907792	5.540399	0.910733	0.978196
APOC1	2.435629	5.470537	5.14037	3.145677	3.472488	1.632268
F12	1.682573	3.347666	3.782409	3.094236	2.253989	3.721373
CRP	0.250962	0	0	0	0	0
HMGCS2	0.111031	0.042644	0.097611	0.042644	7.170326	0.084064
CYP2J2	0.028569	0.042644	0.056584	2.698218	0.163499	0.097611
GCHFR	4.354029	0.847997	4.039138	4.78398	7.192885	4.575312
CYP2C9	0.042644	0	0.014355	0.042644	0.400538	0
RBP4	2.807355	0.464668	1.176323	0.669027	2.819668	0.887525
RBP5	1.169925	1.641546	0.389567	3.032101	1.565597	1.035624
SORD	4.679199	5.434962	4.909293	4.231893	6.740253	4.252476

Supplemented Table S3 continued

Gene	HS766T ACH-000178	MIAPACA2 ACH-000601	PANC1 ACH-000164	SU8686 ACH-000114	SUIT2 ACH-000652	SW1990 ACH-000155
PCSK6	1.790772	2.392317	2.032101	5.402927	6.117072	1.232661
SLC13A5	0.31034	0	0	2.077243	0	0.014355
ASGR1	0.704872	1.773996	0.584963	1.495695	0.871844	0.31034
TM4SF5	0.214125	0	0.124328	0.632268	3.134221	0
ITIH2	0	0.014355	0.014355	0	0	0.014355
TDO2	0	0.432959	0.641546	1.500802	0.650765	0.014355
CPB1	0	0	0	0.432959	0	0
ALDH4A1	4.078097	4.806324	3.563158	2.788686	2.655352	3.181103
ITIH3	0	0.056584	0.028569	0.111031	0	0.201634
C2	0.815575	0.575312	0.575312	2.462052	0.201634	0.863939
CTRB2	0.333424	0.084064	0.070389	0.201634	0	0
ATF5	4.361066	6.84712	7.145066	4.635174	4.393691	5.269033
GP2	0	0	0	0.028569	0.163499	0
CEL	3.119356	1.608809	0.321928	0.903038	0.214125	0.722466
FGG	0	0.056584	0.275007	0.31034	0.042644	0.124328
FGA	0.014355	0	0.137504	0.124328	0	0.137504
FGB	0.042644	0.070389	0.214125	0.176323	0.137504	0.028569
REG3A	0	0	0	0	0	0
REG1B	0	0	0	0	0	0
DHCR7	4.502712	4.917432	7.10842	6.758889	5.830864	2.790772
PC	3.927896	4.164304	3.165108	3.865919	4.216455	2.746313
PNLIP	0	0	0	0	0	0
SERPINA1	2.353323	0.584963	0.400538	5.999549	1.49057	2.513491
ANG	0.948601	1.014355	1.350497	2.827819	2.087463	1.443607
APOC2	0	0	0	0	0	0.056584
