# Supplementary material for: Identification of Genes Associated with Liver Metastasis in Pancreatic Cancer Reveals PCSK6 as a Crucial Mediator
Source: Cancers (Basel). 2022 Dec 30;15(1):241. doi: 10.3390/cancers15010241 (PMC9818395; doi:10.3390/cancers15010241)
Supplement: Supplementary file 1 [file cancers-15-00241-s001.zip › Table S4.pdf]

Supplemented Table S4. Prognostic genes obtained by Lasso Cox regression

Genes	Coefficient of Lasso
AGMAT	0.071011
ALDH4A1	-0.11302
C2	0.162108
CFP	-0.32808
CP	0.034735
F12	-0.06273
GOT1	-0.00017
PCSK6	0.481866
RBP5	-0.03173
TM4SF5	-0.06306
