# Supplementary material for: Identification of Genes Associated with Liver Metastasis in Pancreatic Cancer Reveals PCSK6 as a Crucial Mediator
Source: Cancers (Basel). 2022 Dec 30;15(1):241. doi: 10.3390/cancers15010241 (PMC9818395; doi:10.3390/cancers15010241)
Supplement: Supplementary file 1 [file cancers-15-00241-s001.zip › Table S5.pdf]

Table S5. Cleavage sites of proprotein convertases on target proteins from ELM database

Protein	NCBI Reference Sequence	Accession	Cleavage sites
Kras	NP_004976.2	ELME000100	(K-R-|-X)
		ELME000146	([RK]-X-[hydrophobic]-[LTKF]-|-X)
		ELME000103	(R-X-X-X-[RK]-R-|-X)
Raf	NP_001361187.1	ELME000100	(K-R-|-X)
		ELME000146	([RK]-X-[hydrophobic]-[LTKF]-|-X)
MEK1/2	NP_002746.1/109587.1	ELME000100	(K-R-|-X)
		ELME000146	([RK]-X-[hydrophobic]-[LTKF]-|-X)
ERK1/2	NP_002737.2/002736.3	ELME000100	(K-R-|-X)
		ELME000146	([RK]-X-[hydrophobic]-[LTKF]-|-X)
E-cadherin	NP_004351.1	ELME000100	(K-R-|-X)
		ELME000146	([RK]-X-[hydrophobic]-[LTKF]-|-X)
		ELME000101	(R-X-[RK]-R-|-X)
