# Supplementary material for: Identification of Genes Associated with Liver Metastasis in Pancreatic Cancer Reveals PCSK6 as a Crucial Mediator
Source: Cancers (Basel). 2022 Dec 30;15(1):241. doi: 10.3390/cancers15010241 (PMC9818395; doi:10.3390/cancers15010241)
Supplement: Supplementary file 1 [file cancers-15-00241-s001.zip › Table S6.pdf]

Supplemented Table S6. The intensity ratios of the bands in Western blot

**Repeat 1 of p-ERK in SUIT-2**				
	WT	NC	SC1	SC2
p-ERK	37498.63099	36623.02439	6200.133514	4443.748737
GAPDH	32987.63099	32679.73149	34010.73149	41744.03658
ratio	1.136748225	1.120664789	0.182299329	0.106452301
**Repeat 2 of p-ERK in SUIT-2**				
	WT	NC	SC1	SC2
p-ERK	28521.60155	35161.87363	7078.890873	4818.062446
GAPDH	20554.53048	23865.16652	26271.05382	34556.3381
ratio	1.38760657	1.47335547	0.269455916	0.139426302
**Repeat 3 of p-ERK in SUIT-2**				
	WT	NC	SC1	SC2
p-ERK	41441.72287	38504.28784	6209.669048	4730.819805
GAPDH	29754.20815	26502.26703	28568.92388	41472.08683
ratio	1.392802076	1.452867704	0.217357471	0.114072384
**Repeat 1 of ERK in SUIT-2**				
	WT	NC	SC1	SC2
p-ERK	31399.75231	29046.31728	34695.24621	39438.26703
GAPDH	32160.45942	27073.70206	32909.45942	43737.99495
ratio	0.97634651	1.072860934	1.054263632	0.901693529
**Repeat 2 of ERK in SUIT-2**				
	WT	NC	SC1	SC2
p-ERK	30827.53048	30797.16652	36906.21677	36382.40916
GAPDH	31307.3381	24510.09545	38663.73149	37402.5513
ratio	0.984674276	1.256509449	0.954543583	0.972725333
**Repeat 3 of ERK in SUIT-2**				
	WT	NC	SC1	SC2
p-ERK	29904.13708	26927.75231	36356.31728	41095.16652
GAPDH	35719.48023	31661.70206	38976.80256	37087.90307
ratio	0.837194071	0.850483409	0.93276808	1.108047722
