# Supplementary material for: Identification of Genes Associated with Liver Metastasis in Pancreatic Cancer Reveals PCSK6 as a Crucial Mediator
Source: Cancers (Basel). 2022 Dec 30;15(1):241. doi: 10.3390/cancers15010241 (PMC9818395; doi:10.3390/cancers15010241)
Supplement: Supplementary file 1 [file cancers-15-00241-s001.zip › Table S7.pdf]

Supplemented Table S7. The intensity ratios of the bands in Western blot

**Repeat 1 of p-ERK in MIA PaCa-2**				
	WT	NC	SC1	SC2
p-ERK	36164.30866	36735.28784	16889.12489	3792.334524
GAPDH	33709.70206	33192.95332	30692.85281	39445.68124
ratio	1.072816028	1.106719474	0.550262466	0.096140678
**Repeat 2 of p-ERK in MIA PaCa-2**				
	WT	NC	SC1	SC2
p-ERK	33029.60155	35008.80256	14447.92388	3771.041631
GAPDH	40839.48023	37054.70206	27170.73149	34284.5513
ratio	0.808766453	0.944787048	0.531745856	0.109992445
**Repeat 3 of p-ERK in MIA PaCa-2**				
	WT	NC	SC1	SC2
p-ERK	33927.48023	37088.40916	11582.78175	3664.727922
GAPDH	32700.35891	37277.75231	32273.66043	29106.3381
ratio	1.037526234	0.994920747	0.358892719	0.125908244
**Repeat 1 of ERK in MIA PaCa-2**				
	WT	NC	SC1	SC2
p-ERK	40187.23759	38935.31728	32234.85281	36169.23759
GAPDH	42077.87363	39212.4386	34808.19596	33389.63099
ratio	0.955068166	0.992932821	0.926070769	1.083247599
**Repeat 2 of ERK in MIA PaCa-2**				
	WT	NC	SC1	SC2
p-ERK	38329.01576	41938.02439	31499.66043	31960.75231
GAPDH	43149.53048	43351.3381	34188.07464	29683.21677
ratio	0.888283495	0.967398614	0.921363977	1.076728057
**Repeat 3 of ERK in MIA PaCa-2**				
	WT	NC	SC1	SC2
p-ERK	37529.06602	37455.66043	28145.48885	27702.53048
GAPDH	39829.77312	39339.75231	31150.4386	31326.6518
ratio	0.9422365	0.952107175	0.903534272	0.884311884
