# Supplementary figures and images for: Identification of Genes Associated with Liver Metastasis in Pancreatic Cancer Reveals PCSK6 as a Crucial Mediator
Source: Cancers (Basel). 2022 Dec 30;15(1):241. doi: 10.3390/cancers15010241 (PMC9818395; doi:10.3390/cancers15010241)

A

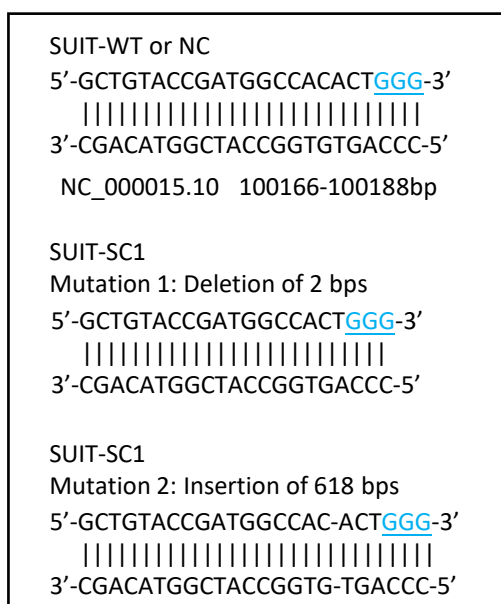

B

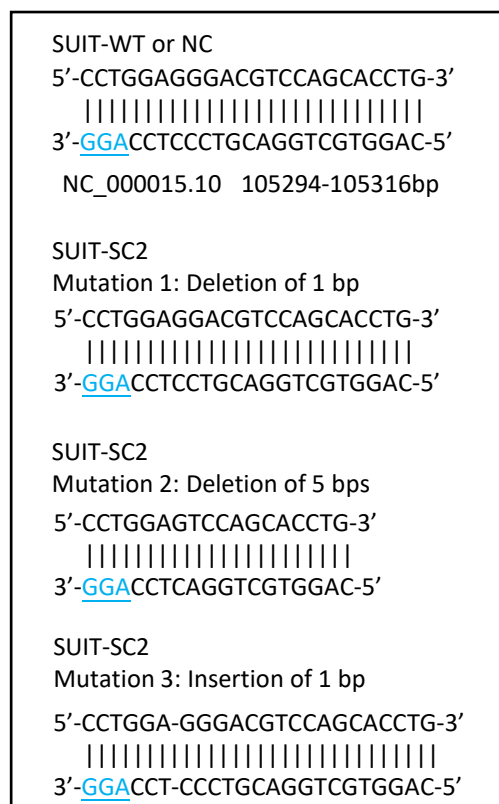

C

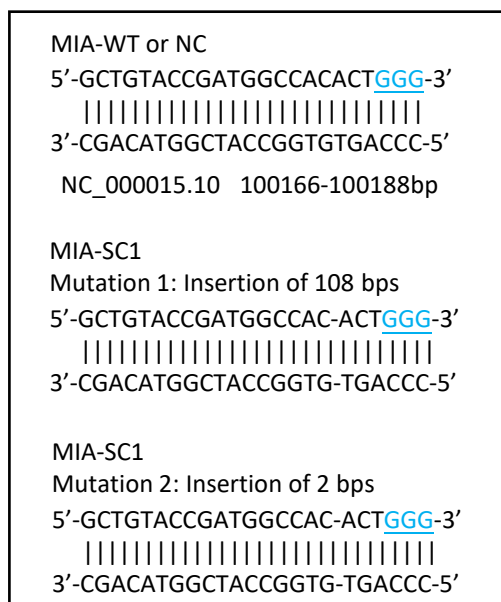

D

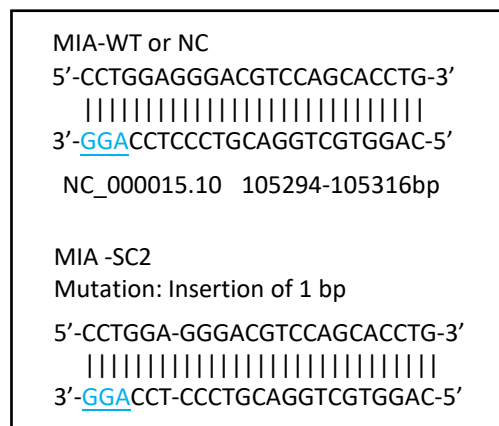

Supplement: Supplementary file 1 [file cancers-15-00241-s001.zip › Figure S1.pdf]

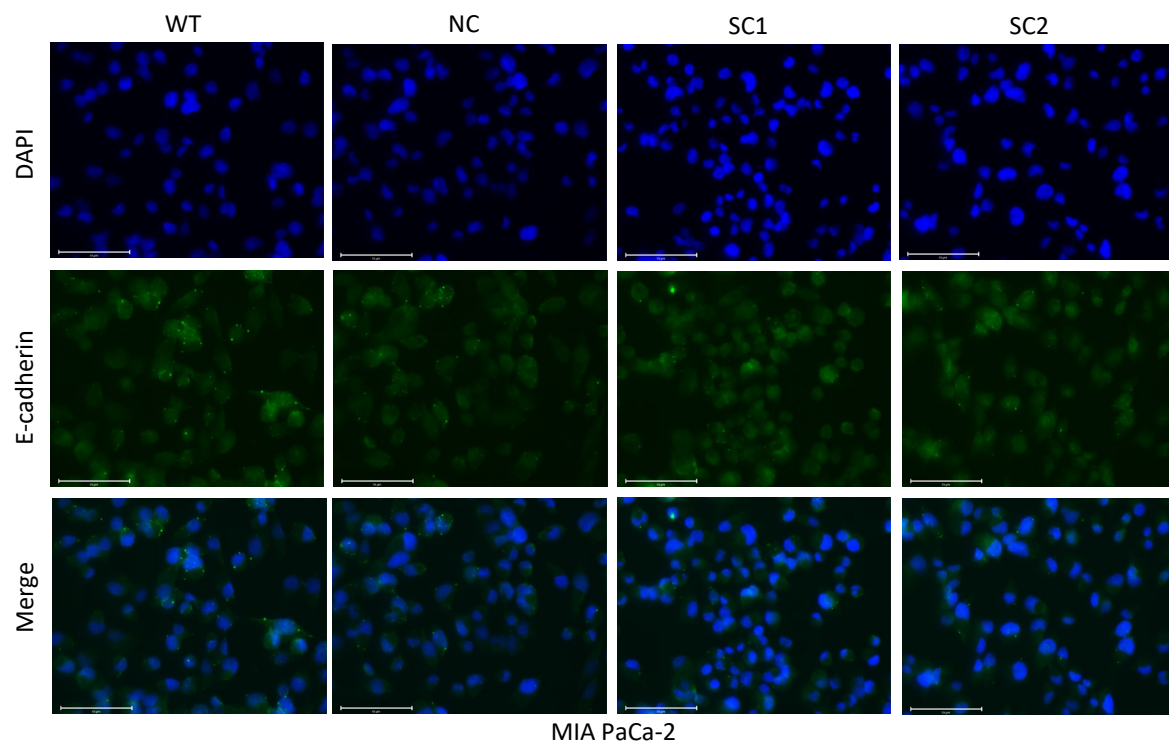

Supplement: Supplementary file 1 [file cancers-15-00241-s001.zip › Figure S2.pdf]

A

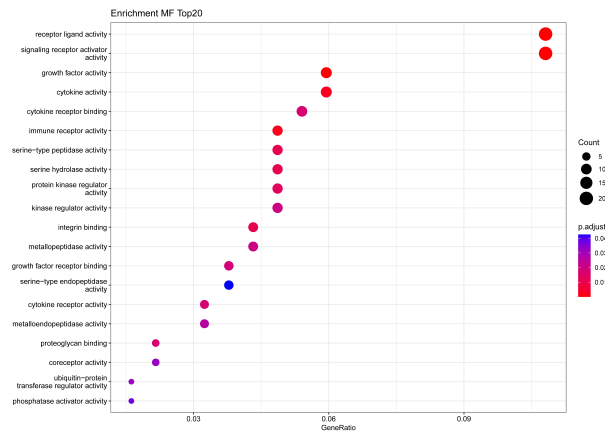

B

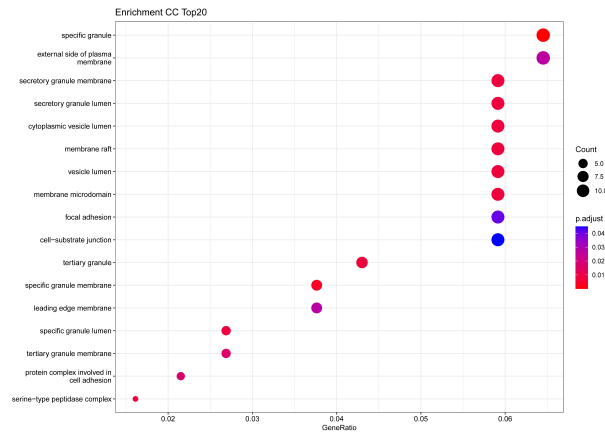

C

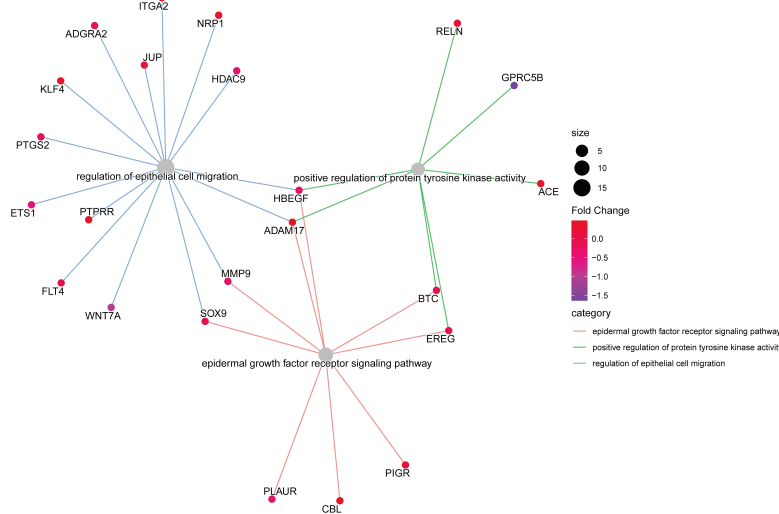

Supplement: Supplementary file 1 [file cancers-15-00241-s001.zip › Figure S3.pdf]
